# Technical considerations in endovascular thrombectomy for distal vessel occlusions

**DOI:** 10.1177/15910199261497569

**Published:** 2026-10-13

**Authors:** Amir Molaie, Nickolas Idrogo, Naoki Kaneko, May Nour, Latisha Sharma, David S. Liebeskind, Gary Duckwiler, Viktor Szeder

**Affiliations:** 1 Neurology, 21767 University of California, Los Angeles, CA, USA; 2 David Geffen School of Medicine, 21767 University of California, Los Angeles, CA, USA; 3 Interventional Neuroradiology, 21767 University of California, Los Angeles, CA, USA

**Keywords:** Distal vessel occlusion, medium vessel occlusion, endovascular thrombectomy

## Abstract

The role of endovascular thrombectomy (EVT) in acute ischemic stroke due to distal vessel occlusions (DVOs) remains to be clarified. Anatomical complexities in the distal vasculature, such as tortuosity and vessel fragility, render technique selection particularly paramount during EVT. Herein, we present two illustrative cases of anatomically similar DVOs treated with two different techniques at a single institution to frame a discussion of important procedural considerations during EVT for DVO.

## Introduction

Endovascular thrombectomy (EVT) is the standard of care treatment for proximal large vessel occlusion (LVO) in acute ischemic stroke (AIS).^1^ However, the optimal management of distal vessel occlusions (DVOs), which comprise up to 25–40% of AIS cases,^2,3^ remains an open question. Recent advancements in thrombectomy technology, including the development of smaller and lower-profile devices, have improved the ability to reach and treat DVOs using various techniques. Despite this, recently published randomized controlled trials (RCTs) concluded that DVO EVT does not confer a clinical benefit over medical management,^4,5^ resulting in American Heart Association guidelines recommending against EVT in this population.^6^ However, these findings are not considered definitive for all patients, in light of concerns raised regarding patient selection, limited EVT success, technique requirements, workflow delays, and outcome measurements, among others.^7^ And entering 2026, two trials were announced with suggestions of clinical^8^ and radiographic^9^ benefit in certain subpopulations: ORIENTAL-MeVO reported improved functional outcomes in patients with moderate to high National Institutes of Health Stroke Scale (NIHSS),^8^ and DISTALS reported successful radiographic improvement using the Tigertriever 13 adjustable stent-retriever (*Rapid Medical*, Yokneam, Israel) in vessels ≥1.5 mm,^9^ though we await the full published results for these trials. In the following report, we present two cases of EVT for DVO from a single institution to underscore the importance of technique selection in optimizing outcomes for these challenging lesions.

### Case 1

A 77-year-old female with a history of coronary artery disease and two prior strokes, resulting in a baseline mRS of 3, presented to our institution with aphasia and right-sided weakness. She was last seen well 7 h prior, with a delta NIHSS of 8. Computed tomography head (CTH) revealed mild gray-white differentiation loss in the left frontal operculum, and CT angiography demonstrated a distal M2/M3 occlusion in the precentral artery, with corresponding hypoperfusion (Figure 1A–C). The suspected mechanism was a central embolic source. She was taken for cerebral angiogram, which confirmed a distal M2/M3 occlusion (Figure 1D).

**Figure 1.**
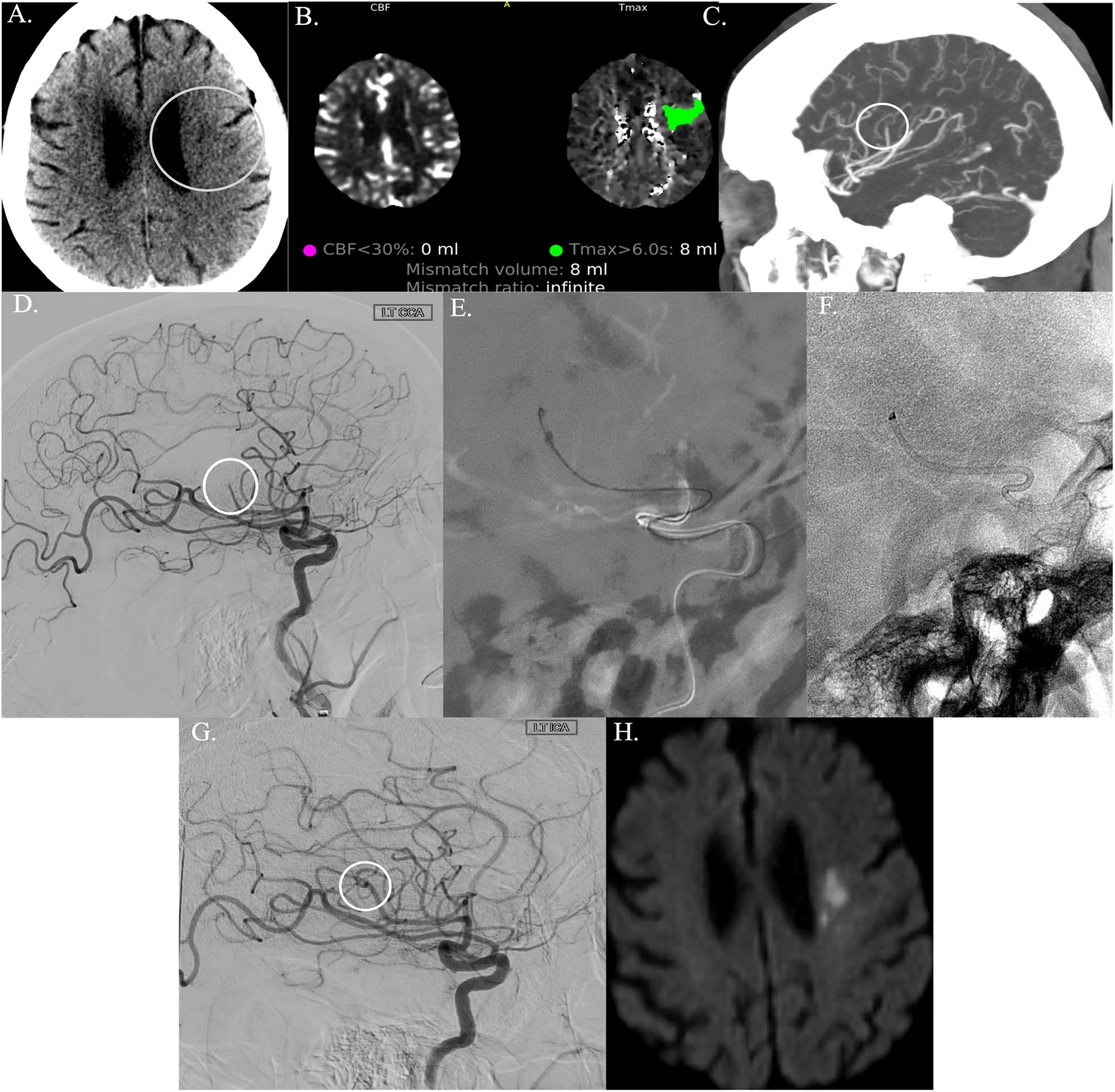
Case 1 imaging and procedural course. (A) CT head demonstrating subtle hypodensity in the left frontal white matter, ASPECTS (Alberta Stroke Program Early CT Score) 9. (B) CT perfusion demonstrating hypoperfusion [*T*_max_ <6 = 8cc, relative Cerebral Blood Flow (rCBF) < 30% = 0] in the region of ischemia. (C) Sagittal CT angiography (CTA) maximum intensity projection (MIP) image demonstrating a distal M2/M3 occlusion in the left precentral artery. (D) Prerecanalization lateral angiogram. (E) Roadmap demonstrating advancement of the microsystem to engage the clot. (F) Spot fluoroscopic image demonstrating the position of the aspiration catheter during clot engagement. (G) Postrecanalization lateral angiogram. (H) Post-endovascular thrombectomy (EVT) diffusion weighted magnetic resonance imaging (MRI) demonstrating scattered areas of diffusion restriction within the left frontal deep white matter.

In light of severe aphasia, risk/benefit discussions with surrogate decision-makers favored EVT. A Zoom 45 aspiration catheter (AC) (*Imperative Care*, Campbell, CA, USA) was assembled over a 0.021″ Trevo Trek microcatheter (*Stryker Neurovascular,* Fremont, CA, USA) and a 0.014″ Synchro Soft microguidewire (*Stryker Neurovascular*, Fremont, CA, USA), which were together placed through a Walrus Balloon Guide catheter (*Q’Apel Medical*, Fremont, CA, USA). The microsystem was advanced distally, and with the microguidewire and microcatheter docked just proximal to the thrombus, the Zoom 45 was advanced into the M3 segment to engage the clot (Figure 1E and F). Continuous aspiration was applied to the AC, and manual negative pressure was applied to the balloon guide catheter lumen during withdrawal. After one pass, no thrombus was noted at the tip of the AC; however, full recanalization was achieved on subsequent angiography (Figure 1G). She returned to baseline functional status by discharge.

### Case 2

A 79-year-old male with a history of atrial fibrillation presented with left facial droop, slurred speech, and left hemiparesis, with an NIHSS of 5. Baseline mRS was 4. CTH revealed subtle effacement of gray-white differentiation in the right frontal lobe, and perfusion imaging demonstrated tissue at risk (Figure 2A and B). Therefore, the patient was taken for cerebral angiogram, where a distal right M2/M3 precentral artery occlusion was found (Figure 2C). Risks and benefits of EVT were discussed with family, who opted for treatment.

**Figure 2.**
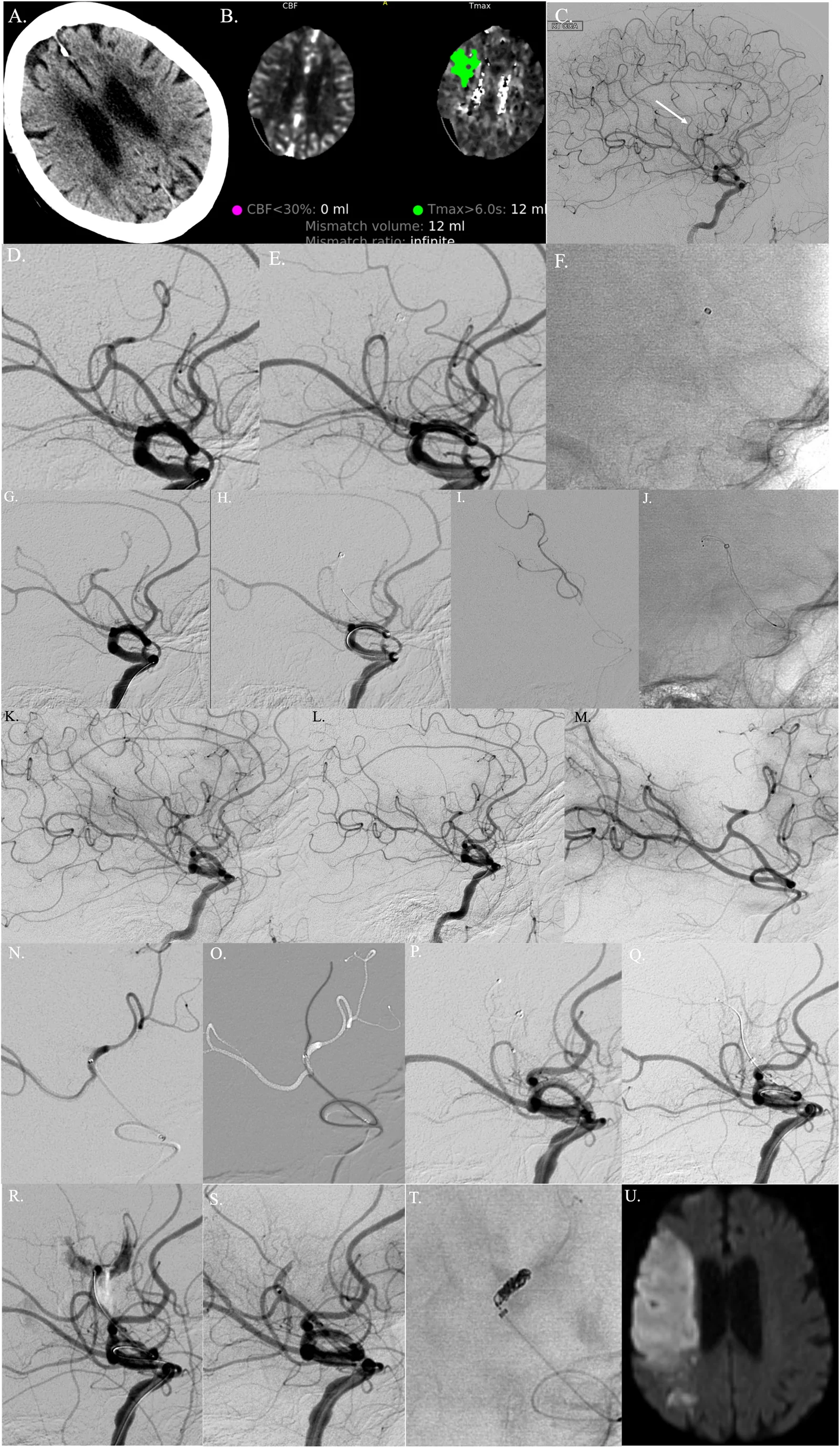
Case 2 imaging and procedural course. (A) CT head revealing subtle loss of gray-white differentiation along with the right middle frontal gyrus and frontal operculum, ASPECTS 8. (B) CT perfusion demonstrating hypoperfusion (*T*_max_ <6 = 12cc, rCBF < 30% = 0) in the region of ischemia. (C) Initial lateral angiogram demonstrating occlusion of the precentral branch. (D–F) Angiograms and spot fluoroscopic image of aspiration catheter position prior to the first pass of aspiration. (G, H) Intraprocedural images depicting crossing the occlusion with the microsystem in preparation for a combined stent-retriever/aspiration pass following unsuccessful aspiration. (I) Superselective microinjection angiogram performed through the microcatheter confirming selection of the precentral branch and microcatheter positioning beyond the thrombus. (J) Spot fluoroscopic image depicting the partially deployed stent-retriever within the aspiration catheter. (K, L) Angiogram revealing transient recanalization, followed by prompt reocclusion. (M) Angiogram after intra-arterial verapamil infusion to confirm absence of vasospasm. (N–R) Fluoroscopic images displaying attempts to recross the lesion with the microguidewire, leading to vessel perforation. (S, T) Tamponade of the perforation with the aspiration catheter, followed by coil embolization. (U) Final MRI demonstrating diffusion restriction in the right middle cerebral artery (MCA) territory.

A first pass of aspiration was attempted using the Vecta 46 AC (*Stryker Neurovascular*, Fremont, CA, USA), tracked over a 0.021″ Trevo Trek microcatheter and a 0.014″ Synchro Support microguidewire, altogether inserted through a Walrus Balloon Guide Catheter. Similar to the preceding case, after the microsystem was docked just proximal to the thrombus, continuous aspiration was applied to the reperfusion catheter, and manual negative pressure was applied to the balloon guide catheter lumen during withdrawal (Figure 2D–F). First-pass aspiration was unsuccessful. Subsequently, a combined approach was employed, wherein the microsystem was advanced beyond the occlusion, and a Trevo NXT 3 × 32 mm stent-retriever (*Stryker Neurovascular,* Fremont, CA, USA) was deployed with the middle of the stent centered around the clot in the M3 precentral branch (Figure 2G–J). The stent-retriever and AC were withdrawn together into the guide catheter. This led to transient recanalization, followed by prompt reocclusion (Figure 2K and L). At this point, 10 mg of intra-arterial verapamil was injected through the catheter to ensure the appearance of reocclusion was not secondary to vasospasm; however, a superselective angiogram did not reveal improvement in vessel caliber, suggesting persistent occlusion (Figure 2M). Another microsystem was assembled with a Headway Duo 156 microcatheter (*MicroVention*, Tustin, CA, USA) and a 0.014″ Aristotle microguidewire (*Scientia Vascular,* Maple Grove, MN, USA), but it was unable to pass the occlusion. After multiple attempts, an angiogram revealed vessel perforation (Figure 2N–R). Heparin was reversed, and the perforation was effectively tamponaded using the Vecta 46 catheter. However, given the continued need for systemic anticoagulation, the decision was made to coil embolize the vessel to prevent rebleeding (Figure 2S and T). The patient's hospital course was complicated by multiorgan failure, and he was eventually transitioned to comfort care.

## Discussion

The anatomical complexity and the relatively mild symptoms from DVO strokes have largely limited the expansion of EVT from LVOs to DVOs. Tortuous access routes consisting of acute angulation through the Sylvian fissure, combined with fragile arterial walls, increase the risk of perforator avulsion, dissection, and vasospasm during EVT.^10,11^ Most commonly, pushing the microwire when the microcatheter is positioned at the vessel wall can lead to vessel perforation, as likely occurred in Case 2. However, unique to DVO clot retrieval, withdrawing a stent-retriever from a distal location can generate a force vector that straightens vessels, increasing the risk of perforator injury.^12,13^ To address this, a variety of combined stent-retriever/aspiration techniques have been described over the last several years, all aiming to maximize successful recanalization while minimizing traction force.^14–16^ However, entering 2025, three major RCTs—DISTAL (Endovascular Treatment for Stroke Due to Occlusion of Medium or Distal Vessels),^4^ ESCAPE-MeVO (Endovascular Treatment of Stroke Due to Medium-Vessel Occlusion),^5^ and DISCOUNT (Mechanical Thrombectomy in Ischemic Stroke With a Medium or Distal Arterial Occlusion)^17^—failed to demonstrate clinical benefit with EVT over best medical therapy in this population, despite varying imaging criteria and technique requirements. Our cases highlight the technical challenges in DVO thrombectomy, which may be crucial as future trials continue to refine patient selection, device innovation, and procedural strategies.

### Clinical considerations

We illustrate two cases of patients with occlusions in similar locations: distally along with the Sylvian fissure, adjacent to the opercular M3 segment.^18^ Clinically, both patients exhibited disabling deficits, though some differences were notable in their premorbid mRS and NIHSS scores. We acknowledge that both patients had premorbid mRS scores which would have precluded enrollment in the recently completed trials of DVO thrombectomy.^4,5^ However, these cases occurred prior to the announcement of the negative DVO thrombectomy trials in 2025, and intervention was pursued after discussion with surrogate decision-makers in light of the patients’ significant deficits. In Case 2, a vessel perforation occurred; because it was detected immediately, the site was coil-packed, leaving the artery effectively in the same occluded state as before treatment. Even so, the patient's initial low NIHSS did not protect him from systemic complications and a poor overall outcome. By contrast, Case 1 shows that when recanalization is completed without procedural injury, neurological function can return to the preocclusion baseline, underscoring the importance of complication-free reperfusion in this population.

### Technique considerations

From a technical perspective, the differences in EVT approaches warrant detailed consideration. Three main techniques are commonly employed: (1) stent retrieval (SR) alone, (2) aspiration alone—often termed direct aspiration first-pass technique (ADAPT)—or (3) a combination approach using both SR and aspiration. The combined approach itself can be subdivided into the *ingestion* technique, that is, pulling the SR through an AC positioned just proximal to the clot, or the *pinching* technique, that is, trapping the clot between the SR and AC, followed by pulling of both the SR and AC together down through the vasculature for proximal ingestion at the guide catheter.^16^

The DISTAL trial reported that the combined approach was the most frequently chosen first-line technique (67.1%) and achieved the highest rate of first-pass and final recanalization, although ADAPT demonstrated the numerically highest rates of good and excellent functional outcomes.^4,19^ The authors noted that symptomatic intracranial hemorrhage (sICH) rates were 7.4% with the combined approach, 8.1% with stent-retriever alone, and 2.8% with aspiration only, and subarachnoid hemorrhage (SAH) rates were similar (30.9% combined, 29.7% SR alone, and 19.4% ADAPT).^19^ In the ESCAPE-MeVO trial, interventionalists were required to use the Solitaire device upfront, though further procedural details remain unreported. A recent large retrospective review compared the efficacy and safety of the three MT techniques in DVO: SR alone demonstrated comparable efficacy to ADAPT, but carried a higher risk of SAH.^20^ This aligns with operator experience, as well as *in-vitro* data, that demonstrated SR alone exerts unfavorable pulling forces across multiple vessel turns when targeting distal clots.^12^ The combination method yielded the highest recanalization rates with the lowest sICH risk, but had the highest SAH risk.^20^ ADAPT exhibited the highest sICH risk, but the lowest SAH risk. It is important to note that this review was conducted prior to the widespread adoption of certain low-profile ACs,^21^ which may account for the elevated sICH risk associated with this technique. Choosing an AC precisely with an optimal catheter-to-vessel ratio has been shown to improve recanalization rates while reducing risk of vessel injury.^22^

It is important to recognize that when discussing the “combined” approach, the distinction between the *ingestion* and *pinching* techniques is rarely reported. However, this difference may be particularly relevant in distal occlusions, where the *pinching* technique is more often employed but exerts greater tug on vessels during withdrawal than *ingestion*.^13^ Therefore, in EVT for DVOs, it may be the case that the combined *ingestion* technique, or aspiration alone, may be preferable to limit vessel traction force, especially if advancements in low-profile aspiration devices improve clot ingestion efficiency. This concept underlies the “Quattro” technique described by Psychogios et al. for M3 occlusions, which employs a quadraxial system to position an AC distally beyond the M2/M3 junction, minimizing the pulling forces transmitted across vessel curves.^14^ That said, it should be noted that *ingestion*, like ADAPT, carries a risk of distal embolization due to clot fragmentation during the suction or ingestion process.^23^ These variables should be explored in future trials. Finally, specialized devices tailored for small caliber vessels can decrease the traction force on distal vessels. In the recently presented DISTALS trial, the adjustable Tigertriever was utilized for every case.^9^ No patients had symptomatic ICH using the Tigertriever 13 device, though one sICH occurred in a patient erroneously treated outside of the protocol with the Tigertriever 17. Information on the precise technique utilized with the Tigertriever (i.e., *ingestion* vs. *pinching* vs. SR alone) remains unpublished; however, the prior consensus recommendations in DVOs advised relaxing/lengthening the device prior to retrieval to avoid wall tension and hemorrhagic complications.^24^

With these principles in mind, our cases highlight both the safety of aspiration-only techniques and the potential risks associated with the combined *pinching* approach. In Case 1, an aspiration-only method was used with first-pass success. In contrast, Case 2 required a technique switch after ADAPT failed to achieve recanalization, possibly due to the tortuosity and angulation within the M3 segment harboring the thrombus, precluding optimal clot engagement. Crossing the clot with a microwire to set up a stent-retriever-plus-aspiration *pinching* maneuver led to vessel perforation. It should also be acknowledged that in such tortuous distal anatomy, when persistent difficulty is encountered in selecting the target branch, a low threshold to abort the procedure should be maintained, given the risks demonstrated herein.

### Future considerations

To improve results in the future, we need to address several points. First, trackability: when ADAPT is preferred but the catheter stalls, neuroendovascular physicians often anchor an SR distally to pull up the AC. Devices that glide to the clot without this extra step would be safest. Second, tortuosity makes ADAPT less effective.^25^ Therefore, aspiration systems capable of clot suction even in sharply curved segments are needed. Third, meticulous microwire control, with frequent roadmap checks and minimal forward force, is essential to prevent perforation when the vessel wall is already under tension.

## Conclusion

These cases illustrate key technical considerations that can potentially inform procedural strategy for EVT in DVO. Given lack of functional benefit, higher procedural risks, and lower reperfusion rates compared to LVOs in three recently completed RCTs, EVT is not considered standard of care for DVOs. However, this underscores the urgent need for further investigation into device and technique innovation to improve outcomes in this challenging subset of AIS.

## Data Availability

Data sharing is not applicable to this article as no datasets were generated or analyzed during the current study.
